# Supplementary material for: Rapid turnover of life-cycle-related genes in the brown algae
Source: Genome Biol. 2019 Feb 14;20:35. doi: 10.1186/s13059-019-1630-6 (PMC6374913; doi:10.1186/s13059-019-1630-6)
Supplement: Supplementary file 3 — Protein alignments, corresponding Gblocks html file and CDS sequences for the lineage conserved gametophyte-biased genes. (ZIP 2109 kb) [file 13059_2019_1630_MOESM3_ESM.zip › Con_GA_alignments/Ec-01_004800.aa_ali.fasta-gb.htm]

Ec-01\_004800.aa.fasta.trans.aa\_ali.fasta


## Gblocks 0.91b Results

Processed file: **Ec-01\_004800.aa.fasta.trans.aa\_ali.fasta**  
Number of sequences: **2**  
Alignment assumed to be: **Protein**  
New number of positions: **486** (selected positions are underlined in blue)

```
                         10        20        30        40        50        60
                 =========+=========+=========+=========+=========+=========+
Ec-01_004800     ------------------------------------------------------------
MSTRG.14482.1    GAGEHSGQHRDMIEKVINVERPGISAYVTCGRDGTIKFWHKGTFVLYRTIRHLDTMKAAY
                                                                             


                         70        80        90       100       110       120
                 =========+=========+=========+=========+=========+=========+
Ec-01_004800     ------------------------------------------------------------
MSTRG.14482.1    EIMITGGKGREAEGSGRRVCLAPVNRAVKSMKSAWVYDIQQMPLSNRIAAACFDRTVSFY
                                                                             


                        130       140       150       160       170       180
                 =========+=========+=========+=========+=========+=========+
Ec-01_004800     -----------------------MVTAKKK----------------------NRGLQDTR
MSTRG.14482.1    DILTGEFVCRIGGLRSSPTCLDCIETGSNQQQVIFGDLDGVLHVWSFGKHFYIPGVQDTR
                                                                       ######


                        190       200       210       220       230       240
                 =========+=========+=========+=========+=========+=========+
Ec-01_004800     EAADLHGNYSLPLHSGAISCVGYVADLNATVTCGVDGKIHFFDLTKRRTTRTFMGHVDRP
MSTRG.14482.1    EAAELNGSYSLPLHSGGVSCVGYVADLNTTVTCGMDGKIHFFDLVKRRVTRTFMGHLDHP
                 ############################################################


                        250       260       270       280       290       300
                 =========+=========+=========+=========+=========+=========+
Ec-01_004800     VFAFAYSKRHRFVVSAGMGRAVVFWNSYTMHVLARRDGHAANVRHCLVDDRSDRVITISA
MSTRG.14482.1    VFAFAYSKRHRFVVSAGMGRSVVFWNSYTMNVLARRDGHAASVRHCLVDDGSDRVITVSA
                 ############################################################


                        310       320       330       340       350       360
                 =========+=========+=========+=========+=========+=========+
Ec-01_004800     DKAVRCWDSATFRCI---------------------------------------------
MSTRG.14482.1    DKAVRCWDSATFRCIQSFRDHRMHRPDDTITAALWDHSRTSLVVAGNRMGVWKNKGAIQS
                 ###############                                             


                        370       380       390       400       410       420
                 =========+=========+=========+=========+=========+=========+
Ec-01_004800     -------------------QIVSGDAMGNVHVWLVETGLLAFRFGKAHGTSKISCMAFDA
MSTRG.14482.1    TKQTHEAPVTAALYNSHFHQVVSGDAMGNVHVWLVETGLLAFRFGKAHGTSKISCMNFDA
                                    #########################################


                        430       440       450       460       470       480
                 =========+=========+=========+=========+=========+=========+
Ec-01_004800     SERRLLTGANDGTVKLWNFNNGKELRLLLPGGNGGGGKAGKKNEEGNERNEVSGVVYCRD
MSTRG.14482.1    SERRLLTGANDGTVKLWNFNNGKELRLFVSGGGPAGG-AVVKLTEGNERNEVSGVVYCRD
                 ##################################### ######################


                        490       500       510       520       530       540
                 =========+=========+=========+=========+=========+=========+
Ec-01_004800     DGKGSDNEVLDYVATCSWDKQVRVFAMESNQEDRRNLAALQQQQQNRHRPLDGGETANAT
MSTRG.14482.1    -GKRSEQEGLDYVATCSWDKHVRVFAMEHGQPP----PAAAQQLQN-HQSVSAGETANVT
                  ##############################                #############


                        550       560       570       580       590       600
                 =========+=========+=========+=========+=========+=========+
Ec-01_004800     VASRVFPGEGPPLHRHH------QGDASGRKGGNSVGGSSRS-----DGEDEGGGNEDTQ
MSTRG.14482.1    IASRVFPEEVRPLQHQHHNNKKHLGHVRGKSRSNRPGGNRKNDYYGEGGDDGGAGPRDAR
                 #################       ##############          ############


                        610       620       630       640       650       660
                 =========+=========+=========+=========+=========+=========+
Ec-01_004800     DRGQGHSADILAIAYCGRQQVATAGFDGRIFIWGTDSGKPKFSMKPSDHDVSQLDEVSSI
MSTRG.14482.1    DPVEGHSADILAIAHCGHQQVATAGFDGRIFVWATDSGKAKFCMKPSDHDVSKLDEVSSI
                 ############################################################


                        670       680       690       700       710       720
                 =========+=========+=========+=========+=========+=========+
Ec-01_004800     GGLTSVAWWMARQRKKDGKVLDSDSV-NSGGYLDRMGGLLAEDASMCHVSWDGRQYFAEE
MSTRG.14482.1    GGLTSVARLMLRQRRERGKNTNADSEEESHGYLDRMESLLAEDASICHVSWDGRQYFAEE
                 #########################   ################################


                        730       740       750       760       770       780
                 =========+=========+=========+=========+=========+=========+
Ec-01_004800     RMGGSDDGSGTAQAANHTTDPDPEGGSAAAADVDENADTAVAGTTAEEVPVVSEEEAKAN
MSTRG.14482.1    RMGGSEDGSGTGQAANVATDPNPEDGSAVEADQTAGL-----------------------
                 ################################                            


                        790       800       810       820       830       840
                 =========+=========+=========+=========+=========+=========+
Ec-01_004800     SSGSVEAKGSAAGVGAAGSDRKGRGGALCPPDQNSPVEPARDEGLAATGGENASPLTSPA
MSTRG.14482.1    ------------------------------------------------------------
                                                                             


                        850       860       870       880       890       900
                 =========+=========+=========+=========+=========+=========+
Ec-01_004800     VLPPSLGADSGGRNSSSSTTGHGANSSKAASATHSADGPAPTSGSASLGPTGKIQETELP
MSTRG.14482.1    ------------------------------------------------------------
                                                                             


                        910       920       930       940       950       960
                 =========+=========+=========+=========+=========+=========+
Ec-01_004800     AAREKGLTSVEKQSKDGRQRQEEEKQQQQQQGVRKEGKGGEESGGRGGGGSRSKGDESSK
MSTRG.14482.1    ------------------------------------------------------------
                                                                             


                        970       980       990      1000      1010      1020
                 =========+=========+=========+=========+=========+=========+
Ec-01_004800     AAAIATAADKEADDGVDGLLENAVSHTVDCLIYIRCHNVLCSGTASGELHVWDASTGAFI
MSTRG.14482.1    ------------------------------------------------------------
                                                                             


                       1030      1040      1050      1060      1070      1080
                 =========+=========+=========+=========+=========+=========+
Ec-01_004800     FGVAARHPNNPGLTAMSTNEGPVERGGGLNHDTPEGLSGRGDGDSLGDGFGVGATRASPA
MSTRG.14482.1    ---------------------------------------------------------APA
                                                                           ##


                       1090      1100      1110      1120      1130      1140
                 =========+=========+=========+=========+=========+=========+
Ec-01_004800     AVRSTTPVAGSRNNLMLFTACEEGFVKTWRLGDITEHVYQTGMRARPKLPRKSRSGRGTK
MSTRG.14482.1    AAEATVDV----------------------------------------------------
                 ########                                                    


                       1150      1160      1170      1180      1190      1200
                 =========+=========+=========+=========+=========+=========+
Ec-01_004800     GSATAGEQVSPLAAGPPAMADSPGTQQTTSLLQPMHQWQAHKTTVTSVQGADYRGKLDLL
MSTRG.14482.1    -----------------------------------------------------------V
                                                                             


                       1210      1220      1230      1240      1250      1260
                 =========+=========+=========+=========+=========+=========+
Ec-01_004800     VTAGDDCYVHVWTIAGAHIGTFGQPTAWSLGSRATWREPTDQALCSAYGHGGAGKEDDEL
MSTRG.14482.1    VDA---------------------------------------------------------
                                                                             


                       1270      1280      1290      1300      1310      1320
                 =========+=========+=========+=========+=========+=========+
Ec-01_004800     SATTTTASTATAAAGEKESKAKTQGGSKHLRRTASKHGKAVTSTTTDGGDEKEPQKGRKH
MSTRG.14482.1    ------------------------------------------------------------
                                                                             


                       1330      1340      1350      1360      1370      1380
                 =========+=========+=========+=========+=========+=========+
Ec-01_004800     HDQRARAAQDRDLEKEREEEAERCQEKRYYIASQNRVQRKLFRGRLSAFCVSRDYYKKET
MSTRG.14482.1    -------------ET---------------------------------------------
                                                                             


                       1390      1400      1410      1420      1430      1440
                 =========+=========+=========+=========+=========+=========+
Ec-01_004800     IQEATQILSETCKKSALHPQPSSSRPVLSKHRITSILRNTYNQVSFQLQLHKHEDVHERA
MSTRG.14482.1    -------------------KPAEEMP----------------------------------
                                                                             


                       1450      1460      1470      1480      1490      1500
                 =========+=========+=========+=========+=========+=========+
Ec-01_004800     EVALTRRKSRRRAKAAASSTSHQHHPHHPRNPGGTAETNRPSSMTLTAATAPSTPHTPAD
MSTRG.14482.1    ------------------------------------------------------------
                                                                             


                       1510      1520      1530      1540      1550      1560
                 =========+=========+=========+=========+=========+=========+
Ec-01_004800     TATPRTVSRATGVEGGRSGSATPAEDGTVPRTDGHGGGGSGGHGSRAMPGTKAARGTDTI
MSTRG.14482.1    ------------------------------------------------------------
                                                                             


                       1570      1580      1590
                 =========+=========+=========+=====
Ec-01_004800     SKDREGNVRPAAPVDPPSSSATSGRRFRRPSAAMG
MSTRG.14482.1    -------------------------------AIPE
```

```
Parameters used
Minimum Number Of Sequences For A Conserved Position: 2
Minimum Number Of Sequences For A Flanking Position: 2
Maximum Number Of Contiguous Nonconserved Positions: 8
Minimum Length Of A Block: 10
Allowed Gap Positions: None
Use Similarity Matrices: Yes
```

```
Flank positions of the 9 selected block(s)
Flanks: [175  315]  [380  457]  [459  480]  [482  511]  [528  557]  [565  578]  [589  685]  [689  752]  [1079  1088]  

New number of positions in Ec-01_004800.aa.fasta.trans.aa_ali.fasta-gb:  486  (30% of the original 1595 positions)
```
